# A Shallow Autoencoder Framework for Epileptic Seizure Detection in EEG Signals

**DOI:** 10.3390/s23084112

**Published:** 2023-04-19

**Authors:** Gul Hameed Khan, Nadeem Ahmad Khan, Muhammad Awais Bin Altaf, Qammer Abbasi

**Affiliations:** 1 Department of Electrical Engineering, Lahore University of Management Sciences (LUMS), Lahore 54792, Pakistan; nkhan@lums.edu.pk (N.A.K.); awais.altaf@lums.edu.pk (M.A.B.A.); 2 Engineering and Design Department, Western Washington University, Bellingham, WA 98225, USA; 3 Communications Sensing and Imaging Research Group, James Watt School of Engineering, University of Glasgow, Glasgow G12 8QQ, UK; qammer.abbasi@glasgow.ac.uk

**Keywords:** autoencoder, EEG classification, epilepsy, seizure detection

## Abstract

This paper presents a trainable hybrid approach involving a shallow autoencoder (AE) and a conventional classifier for epileptic seizure detection. The signal segments of a channel of electroencephalogram (EEG) (EEG epochs) are classified as epileptic and non-epileptic by employing its encoded AE representation as a feature vector. Analysis on a single channel-basis and the low computational complexity of the algorithm allow its use in body sensor networks and wearable devices using one or few EEG channels for wearing comfort. This enables the extended diagnosis and monitoring of epileptic patients at home. The encoded representation of EEG signal segments is obtained based on training the shallow AE to minimize the signal reconstruction error. Extensive experimentation with classifiers has led us to propose two versions of our hybrid method: (a) one yielding the best classification performance compared to the reported methods using the k-nearest neighbor (kNN) classifier and (b) the second with a hardware-friendly architecture and yet with the best classification performance compared to other reported methods in this category using a support-vector machine (SVM) classifier. The algorithm is evaluated on the Children’s Hospital Boston, Massachusetts Institute of Technology (CHB-MIT), and University of Bonn EEG datasets. The proposed method achieves 98.85% accuracy, 99.29% sensitivity, and 98.86% specificity on the CHB-MIT dataset using the kNN classifier. The best figures using the SVM classifier for accuracy, sensitivity, and specificity are 99.19%, 96.10%, and 99.19%, respectively. Our experiments establish the superiority of using an AE approach with a shallow architecture to generate a low-dimensionality yet effective EEG signal representation capable of high-performance abnormal seizure activity detection at a single-channel EEG level and with a fine granularity of 1 s EEG epochs.

## 1. Introduction

Epilepsy is the second most probable neural disease of the brain according to the WHO, affecting more than 50 million people worldwide. It is the most severe chronic neurological disorder of the central nervous system and is characterized by repeated unprovoked seizures [1,2]. Epileptic seizures can cause severe upheaval in the emotions, behavior, and consciousness of patients and can lead to severe injury or even death [3,4].

Electroencephalograms (EEG) are a conventional and relatively inexpensive method for the detection and diagnosis of epilepsy [5]. Epileptic seizure detection in EEG signals involves recognizing brain abnormalities that distinguish normal activities (non-ictal) from unusual ones (seizure) [6]. Seizures are usually detected by the visual analysis of EEG signals by neurologists [6]. However, EEG signals demand neurology professionals for the visual assessment of long-duration EEG recordings, which is extremely laborious [7]. The accurate detection of these disorders requires us to develop an efficient algorithm to capture brain abnormalities associated with epileptic seizures in the scalp EEG signals to support clinicians. This will help to minimize human efforts and error [8].

Various algorithms have been developed in recent studies on automatic seizure detection using multi-channel [9] and single-channel EEGs [10]. Hardware implementations for real-time seizure detection in wearable EEG devices are also advancing fast [11]. The constant monitoring of epileptic patients is indispensable to prevent the serious outcomes that can be caused by seizures. In consequence of these requirements, wearable EEG devices integrated with epileptic seizure detection algorithms have become essential for health management systems. Moreover, these methodologies have led to the proposal of patient-specific algorithms [12] and patient-independent methods [13].

The main contribution of our paper is that it proposes a low-complexity, high-performance seizure detection approach that is based on single-channel analysis. In contrast to other approaches, our approach allows abnormal seizure activity identification at the channel level. It out-competes existing approaches not only in these respects but also due to its suitability of use in wearable devices by minimizing the number of channels. Furthermore, our hybrid approach does not involve the use of traditional features, which are laborious to select. In contrast, it uses the low-dimensional encoded representation of the signal generated by training a shallow autoencoder (AE) by minimizing the reconstruction error of the decoded signal. This encoded representation is effectively classified by a conventional classifier. We report two algorithms based on our experiments: (a) one with the best classification performance compared to the reported methods and (b) another one with the best performance compared to single-channel approaches but of the lowest reported computational complexity. We have presented extensive results on two datasets using different AE hidden sizes with several classifiers. Furthermore, our approach successfully compiles most of the powerful features of the different approaches into a single approach.

The following section describes the recent advancements in seizure detection. The design methodology of the work is presented in Section 3. Section 4 discusses the algorithm of the proposed method. A brief overview of the experimental setup along with the method’s classification performance and computational complexity is presented in Section 5. Concluding remarks are addressed in Section 6.

## 2. Related Works

**Figure 1:**
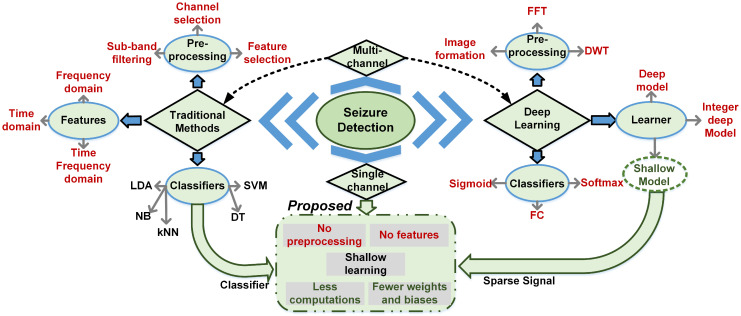
A general overview of seizure detection methods.

Numerous algorithms have been proposed in the literature for classifying EEG data as epileptic or non-epileptic. Figure 1 depicts a general overview of the main components of these methodologies. Seizure detection methods can be mainly categorized into two classes. The first one relates to conventional classification methods computing statistical features along with machine learning tools for classification. The second one corresponds to deep learning models using artificial neural networks. Both multi-channel and single-channel approaches are reviewed. A short review of recent preprocessing methods is also provided below.

### 2.1. Preprocessing Methods

EEG signals are preprocessed to improve the quality and reliability of the data. Preprocessing techniques are used to remove or reduce the artifacts and enhance the underlying neural activity in the signal. This may involve filtering out unwanted frequencies, removing or correcting for artifacts, and re-referencing the signal to a common reference electrode. Moreover, the preprocessing steps may include segmentation of the signal into epochs, artifact rejection, baseline correction, and normalization of the data. EEG signals are often contaminated with various artifacts, including muscle activity and eye movements, which can make it difficult to extract meaningful information from the signal [14]. An EEG signal artifact detection methodology was developed in [14]. This study offers a novel technique for automatically identifying muscle and eyeblink artifacts from EEG data using a kNN classifier and subsequently eliminating them using a long short-term memory (LSTM) network. A baseline removal approach was developed in [15]. The suggested approach in this article is the InvBase method, which aims to eliminate the baseline power prior to feature extraction to ensure that the extracted features remain consistent across different subjects. Moreover, polar projection- and fast Fourier transform (FFT)-based data transformation have also been addressed as a preprocessing step for EEG classification [16]. Normalization of the EEG signal before using it for abnormal activity identification has also been proposed in the literature [17]. Additionally, some existing approaches use raw EEG data directly without any preprocessing [18,19].

### 2.2. Classification Methods—Conventional

Overall, the detection of epileptic seizures using conventional techniques has three subdivisions, as shown in Figure 1. Most of these techniques compute statistical features from multi-channel EEG data [4,8] represented in the time domain, frequency domain or time-frequency domain. These features include min-max histogram [8], energy, entropy, and the variance coefficient, along with others [20]. Signal enhancement methods, such as common spatial patterns (CSP), and dimensionality reduction techniques, such as principal component analysis (PCA), are extensively used to fine-tune the features [6]. Spectral and temporal feature computation using scattering transform (ST) and discrete wavelet transforms (DWT) have also been addressed for seizure detection [21,22]. Ramanujan periodic subspace (RPS) [23] and empirical mode decomposition (EMD) [24] have also been proposed. The selection of EEG electrodes through adaptive ranking or manual choice has also been proposed for performance improvement [2]. The optimal selection of the customized features along with feature ranking has also been discussed in previous approaches to enable the classifier to demonstrate better learning [4,8]. This phenomenon also adds complexities to the system and demands more resources. Seizure detection based on the direct use of compressively sensed EEG data was proposed in [25]. However, the authors used Lomb–Scargle periodograms (LSP) to extract the spectral energy features of the compressed data, which adds extra computations to the process. A generalized autoregressive conditional heteroscedasticity (GARCH) model was proposed in [26] to determine the hidden properties in epileptiform EEGs. Confidence intervals were created using the delta and asymptotic method to compare the half-lives to determine the volatility attributes of seizure and non-seizure intracranial EEG data.

These features can be used as input for machine learning classifiers to categorize the data into different classes. Machine learning algorithms such as kNN, SVM, linear discriminant analysis (LDA), quadratic discriminant analysis (QDA), decision tree (DT), random forest (RF), and the naive Bayes (NB) classifier are mostly used to categorize the data.

The above approaches vary in number of channels from 8 to 23 [27,28]. Various single-channel approaches have also been proposed. Ref. [17] used compressively sensed EEG data directly for classification, but segment-level results were not provided. Ref. [25] performed seizure detection based on partially reconstructed EEG data from compressively sensed data using orthogonal matching pursuit (OMP). The approach utilizes the energy features of the reconstructed signal for two different compression ratios. Results were presented for signals with compression ratios of 0.05 (1:20), 0.1 (1:10), and 0.2 (1:5). Though the involved compression ratios are high, the reported classification performances are low. The computational cost is also high. Wavelet-based features of single-channel EEG data are presented in [22,29]. However, the classification performance of all these approaches is quite low, and significant improvements are essential for real-life applications.

Conventional methods have also been implemented in hardware. Both multi-channel and single-channel approaches have been adopted. Examples of hardware modules based on multi-channel approaches are [11,30]. They use statistical features, non-linear computations [11], and wavelets [30] with considerable computational cost. A single-channel epileptic seizure detection ASIC design was discussed in [10]. Ten sub-band FIR filters with a band energy feature were used for an SVM classifier. A significant area reduction was reported, along with low power consumption; however, the classification performance needs to be improved.

Finally, it should be noted that wearable health devices require low computational complexity both in terms of power consumption and computing resources. Existing methods are still computation- and resource-hungry. Moreover, due to the highly nonstationary nature of EEG data, conventional statistical features are unable to capture their actual nature. This may lead to missing the non-stationary property in epileptic EEGs and produce suboptimal recognition. More work is required on algorithms that are reliable, computationally light, and hardware-friendly to improve wearable health systems [31]. In addition, some approaches use patient-specific features [20,24,32] and might be difficult to generalize to other patients’ data. The actual performance of these algorithms is a major concern in real-life health monitoring systems.

### 2.3. Classification Methods—Neural Networks

The detection of epileptic seizures using deep learning models has evolved rapidly in recent times. Deep learning models such as convolutional neural network (CNN) architectures, stacked autoencoder (SAE), and convolutional autoencoder (CAE) developed for classifying EEG data result in high-accuracy systems [5,9,16]. These methodologies apply data preprocessing or use raw EEG data directly for model training [5]. AE or neural networks can be trained to compute weights and biases to obtain unique parameters from EEG data. Then, classification is carried out by adding a softmax layer at the end of the network. These models are trained based on classification metrics. An integer CNN model was proposed in [33] for hardware-friendly computations. Quantized deep neural networks (QDNNs) for low-power embedded applications have also been developed [34]. Furthermore, deep transfer learning techniques for classifying EEG data have been established in the literature [35]. A combination of four different CNN models and transfer learning with a ResNet50 model was developed in [35]. Deep network architectures provide satisfactory results by paying the penalty of requiring immense resources, which is not feasible for the development of wearable health systems. Architectures comprise many computational layers, with mathematical operations increasing exponentially alongside the increase in the number of layers. Secondly, the weights and biases of each layer have to be saved, which results in the utilization of more resources. A major drawback in many neural network approaches is their computational complexity, especially when deep architectures are employed. For this, shallow networks instead of deep architectures are suggested.

Hybrid architectures have been proposed based on generating some encoded representation followed by classification using a conventional machine learning approach. The detection of epileptic seizures utilizing compressively sensed EEG signals has been extensively reported in the literature [17,25,36]. In these studies, there are both single- and multi-channel approaches. In [17], a denoising sparse AE (shallow encoder)-based hybrid approach was adopted for ictal EEG classification, where the classification of encoded representation was carried out through logistic regression. The shallow AE architecture supports developing a system with fewer computations and less resource utilization as compared to deep networks. However, the preprocessing of the input signal was carried out through z-score standardization and by adding random noise to it, which increased the processing. The AE structure is 256-200-256, providing 1.28 CR. This compression ratio is not large enough. Thus, no EEG epoch classification has been done. Moreover, it uses the BONN dataset, which is not a large dataset.

It should be generally noted that the reviewed approaches have demonstrated results on a single dataset. Finally, none of the multi-channel approaches provide the classification of EEG epochs at the individual channel level. Their classification is based on the collective analysis of channels involved.

## 3. Materials and Methods

### 3.1. Datasets

In order to evaluate the proposed seizure detection method, we used a publicly available PhysioNet scalp EEG database, named CHB-MIT, provided in [37]. This dataset was collected at the Children’s Hospital Boston. It contains 916 h of recordings of intractable generalized seizures from pediatric subjects. Recordings were collected from 22 subjects grouped into 23 cases and sampled at 256 Hz. Ref. [38] can be referenced for tabulated information on the subjects involved.

We also used another publicly available EEG dataset provided by the University of Bonn [39]. The dataset comprises 5 different sets of single-EEG-channel recordings, named A, B, C, D, and E. A and B correspond to scalp EEGs of healthy subjects, whereas C, D, and E are intra-cranial EEGs. Each set has 100 EEG files with generalized seizures. Details of the database are provided in [36]. As many researchers have used this dataset [23,38,40,41,42], we have also included this dataset for the purpose of comparison.

### 3.2. Proposed Method

#### 3.2.1. Design Methodology and Considerations

In the previous section, we reviewed various conventional and neural network methods for epilepsy detection, along with their pros and cons. As reviewed earlier, both multiple- and single-channel approaches have been proposed. This depends on the targeted application. Most of these approaches that are high performance are multi-channel and computationally intensive. Neural network architectures generally involve deep architectures, while conventional approaches involve complex feature extraction stages.

Compact wearable devices, wireless sensor networks, and associated data processing are of increasing importance for diagnosis and patient monitoring. One should also consider minimizing the number of channels for the wearing comfort of ambulatory patients. Many hardware implementations have, therefore, adopted designs utilizing a low number of EEG channels and even a single channel. The application of such systems can be seen with reference to the extended diagnosis of epilepsy (generalized or focal seizures) and in monitoring the effectiveness of the treatment. Monitoring, recording, and marking seizure activity in one or a few channels in ambulatory patients at home can augment the status information obtained from the full-fledged EEG captured at the hospital. In such cases, the choice of these few monitoring locations will be the prerogative of the treating neurologists. This decision can be made by the neurologists after diagnosing the epilepsy type and observing abnormal wave complex activity in the EEG at different scalp locations.

The current focus of our work is to propose a computationally light and hardware-friendly approach that can analyze a single EEG channel with high accuracy to detect seizure activity. Most of the existing approaches employ multiple channels of EEG to extract a group feature vector and label the EEG epoch as seizure (ictel) or non-seizure (non-ictel) activity. Such an approach does not localize the evidence of the abnormal seizure activity to the channel level. Hence, a neurologist has to accomplish this on their own to accept or reject the automatic classification result. Furthermore, an approach based on processing a channel individually has a better potential to be scalable with respect to the number of channels, allowing its deployment in devices using one, few, or all EEG channels. For a multi-channel detection framework, this will mean processing the given channels separately as proposed and then assembling the individual detection results for a final global decision. This, however, is not discussed in this work. Furthermore, the scope of the current work is limited to achieve low algorithmic computational complexity but does not cover hardware implementation. This could be undertaken in the future.

As discussed earlier, AE models have been proposed in the recent literature [9,17] for epileptic seizure detection. Shallow AEs (involving a single layer) have been shown in earlier works to be successful in the generation of effective encoded samples (as features for classification) [17]. They learn the essential representations of data from a number of sample sets [17]. They have been extensively utilized in areas such as image de-noising [43], feature learning [44], and data modeling [45], etc. For any considered dimension of the latent space, ref. [45] reports that the best performance for unsupervised learning is obtained with AE for image databases.

This work experiments with a trainable hybrid approach involving a shallow AE and a conventional classifier. A shallow AE, instead of a deep neural architecture, is considered to provide an encoded representation of the input signal. A shallow AE brings a smaller number of parameters to train and store and are computationally efficient. Theoretical evidence about the fact that shallow AE models indeed can be used as a feature learning mechanism for a variety of data models has been proved in [45]. A similar mechanism has been adopted in this work to develop our model taking into account our data. Conventional classifiers have been used to realize seizure detection or classification on this encoded representation of the input signal. An effective lower-dimensional encoded signal (smaller feature vector) in turns reduces the computational complexity and training effort along with the improvement in classification performance.

The proposed system involves a two-stage training process that involves training the AE first and then training the classifier. An encoded representation of the signal is trained in the first stage of the training process involving both the encoder and the decoder. The decoder reconstructs the signal to its original shape, and the objective of training is to minimize the reconstruction error. In the second stage, the trained encoded signal samples are used to train the classifier. Various conventional classifier models have been experimented on to see possible compromises in computational costs and classification performances suitable for different scenarios. The tested classifiers include kNN, SVM, DT, QDA, NB and softmax. The classification results of these models are discussed in the coming sections.

For determining the optimal design, multiple AE hidden sizes and different conventional classifier models have been experimented with. Analysis has been conducted on multiple hidden sizes to find the right balance for a shallow network that is large enough to capture the actual nature of the data and yet small enough to run faster as compared to other network architectures in the literature.

A completely training-based approach is suitable for both the ease of initial development and re-training for introducing patient specificity and adaptability to provide enhanced performance. The training or re-training is essentially more computationally intensive than test or in-operation-mode computation. However, as the algorithm training is usually done offline in the initial training phase or re-training phase, it is the computational effort in the testing (or operational) mode that matters most with respect to the minimization of energy and the computational time of the device. A detailed analysis and comparison of the computational cost in the testing phase of the model will be provided in the upcoming sections.

A short review of the theory of shallow AEs is provided as follows.

#### 3.2.2. Shallow Autoencoder

An autoencoder is an unsupervised learning technique for neural networks that learns efficient data representations (encoding) by training the network to ignore signal “noise”. It learns to transform the data from a high-dimensional space to a lower-dimensional space. They work by encoding the data to a 1-D vector, which can then be decoded to reconstruct the original data. As compared to principal component analysis (PCA), autoencoder works for both linear and non-linear surfaces, whereas PCA only works for linear surfaces.

**Figure 2:**
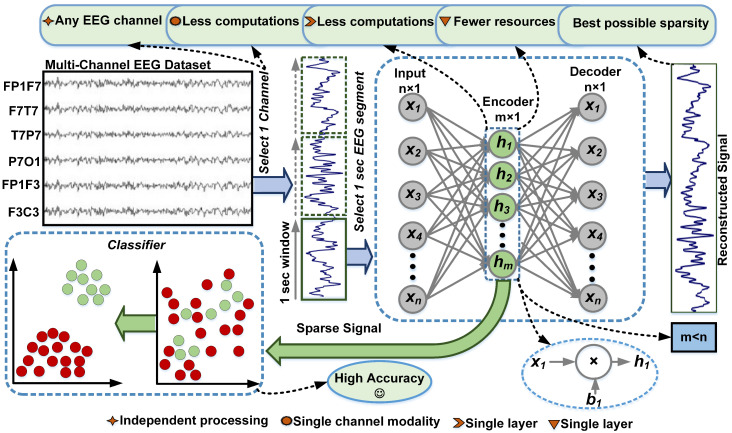
Workflow architecture of the proposed seizure detection method.

A shallow AE with a single hidden layer comprised of *n* neurons in the input/output layer and *m* hidden neurons is developed with m<n, as shown in Figure 2. The encoder layer is evaluated by sharing the weights $W^{E}\in R^{n\times m}$ and bias vectors $b\in R^{m}$. The decoder layer reconstructs the signal with the weights $W^{D}\in R^{m\times n}$ and bias vectors $b\in R^{n}$. These weights and biases of the model are calculated for the signal reconstruction error instead of the classification results. The scaled conjugate gradient (SCG) method was designated to update these weights and bias values. Ref. [44] averts the decoder bias, but we consider it an essential part of our model. The encoding process for an input EEG signal $x\in R^{n}$ is modeled as:

(1) $$y=f(W^{T}x\;+\;b)$$

where f(.) represents the activation function in the encoder neurons. The choice of the activation function usually varies with different data [44]. However, linear activation aids in developing a system with a low computational cost as compared to non-linear functions. Therefore, we selected linear activations for the encoding and decoding processes. The saturating linear transfer function (Satlin) [46] for the encoder activation function is given as:

(2) $$satlin\left(x\right)=\left\{\begin{array}{cc} -1 & x<-1\\ 0 & -1⩽x⩽1\\ 1 & x>1 \end{array}\right\}$$

The decoder layer reconstructs the encoded vector $y\in R^{m}$ to its original form, as follows:

(3) $$\hat{x}=W^{D}y$$

The transfer function to compute the decoder layer’s output is a linear function (Purelin) f(x)=x. These functions are carried out on each sample of the signal with the mean square error (MSE) loss function. MSE is selected in the training phase of the AE to compare the original and reconstructed signal, and the AE model is trained until the loss function achieves its minimum possible value. Moreover, during the training phase, the $L_{2}$ regularization parameter is used to avoid model over-fitting. The encoded representation of the signal is achieved through encoding, and the reconstruction of the signal to its original form is achieved through the decoder. The decoding process is added to ensure the best possible encoded representation of the data.

#### 3.2.3. Processing Architecture

Figure 2 depicts the basic processing architecture of the proposed methodology. The process starts with EEG data segmentation. EEG signal epochs of 1 to 30 s are usually adapted for different seizure detection methods in the literature [47]. These durations are short enough to capture the dynamics of seizure activity while still being long enough to provide sufficient data for analysis. Shorter epochs in the stated range can enable the detection of seizure onset with low latency and can easily provide instances that are artifact- and noise-free. Thus, a 1 s non-overlapping EEG epoch is selected in this paper for accurate marking at fine granularity. This determines the system latency is 1 s as it detects the seizure for each second of EEG recording. An AE with a single hidden layer processes each 1 s EEG epoch to produce its encoded representation, as shown in Figure 2. The input signal is encoded using the encoder layer to obtain an encoded vector. Once the AE is trained, the decoder layer is discarded, and only the encoder layer is applied for the encoded representation of the data, which is then used as an input to the classifier.

The steps necessary to process a 1 s EEG signal epoch are as follows. First, the database of our choice is at a sampling rate of 256 samples/s. Therefore, the input layer consists of a length of 256. The encoder compresses the input signal to 64 samples, whereas the decoder layer reconstructs the signal to its original length of 256, providing a compression ratio of 4 in our core experiment. Multiple experiments have been carried to determine the optimum value of the AE’s hidden size to obtain the best performance. However, for large values of the AE’s hidden size, the algorithm tends to learn very local features of the input data, which reduces the classification performance.

Table 1 encapsulates different lengths of encoded representations of EEG signals used in this paper, along with the corresponding data reduction ratios. The analysis of multiple AE hidden sizes is provided to evaluate the classification performance along with the computational complexity of the system. Therefore, the number of arithmetic operations (AO) for the AE and classifier are also presented to evaluate the exact computations with corresponding classification results. Our primary experiment deals with an AE hidden size 64, providing 75% diminution in the data. However, other levels are also part of our analysis and produce comparable results, even applying more than 90% data reduction. Consequently, the user has the choice to select any AE hidden size and achieve the corresponding data reduction among the listed options.

In order to categorize the data as epileptic or non-epileptic, the encoded representation of EEG epochs from the output of the encoder is used. Various machine learning algorithms are used for classification, such as kNN, SVM, DT, QDA, NB, and softmax layers. Adding a deep neural network (DNN) model may provide good results, but its computational cost would be quite high.

**kNN:** We selected 10 nearest neighbors according to their Euclidean distance for the kNN classifier. Weighted kNN is used to handle uncertain conditions with a squared inverse distance weight kernel. In order to encapsulate the uncertain outliers and to deal with incomplete and inconsistent information existing in the features, the exhaustive neutrosophic set was used to determine the decision criteria [48].

**SVM:** For the SVM classifier, multiple kernel functions were tested. The impact of various kernel functions on SVM characteristics is highly variable. In this paper, we used the Gaussian kernel with random search. The hyper-parameters were randomly sampled from a specified distribution, and the model was trained and evaluated for each set of sampled hyper-parameters. An optimized hyperplane in kernel space was generated by a Gaussian function of kernel scale 2 and kernel offset 0.1. Training instances are separable for these parameters and yield optimal results. The feature space that training data is mapped to is determined by the kernel width. The optimal kernel scale value is the one that yields the best performance on the test set.

**QDA:** QDA is similar to LDA but without the assumption that the classes share the same covariance matrix, i.e., each class has its own covariance matrix. In this case, the boundary between classes is a quadratic surface instead of a hyperplane.

**DT:** DT is listed as the least computationally expensive classifier [49]. To find the best split, we used the ’exact search’ algorithm with a tree of 159 nodes and the ’gdi’ split criterion.

**NB:** NB is based on Bayes’ theorem derived from the conditional probabilistic theory with an assumption of independence among predictors [4]. In simple terms, an NB classifier assumes that the presence of a particular feature in a class is unrelated to the presence of any other feature. We used the Gaussian kernel function with a normal distribution for this classifier.

**Softmax:** Softmax layers are often used as the last layer of neural networks trained for data classification [5]. We trained our softmax layer over an encoded vector in the same manner as previously listed classifiers. The ’binary cross-entropy’ function was used with the SCG algorithm to update the weight and bias values.

## 4. Results

### 4.1. Experimental Setup

The proposed algorithm was implemented using MATLAB 2018. The experiments were performed on a system with an Intel Core i7 processor at 3.20 GHz and with 24 GB memory.

In the case of the CHB-MIT database, around 916 h scalp EEG recordings containing 185 seizure events were adopted in this study. About 5 recordings (1 s duration EEG epoch), including randomly selected seizure and non-seizure events, were used as the training set. The seizure and non-seizure ratio is 40% and 60%, respectively. The reason for this choice is the fact that the dataset comprises long hours of EEG recordings with rare seizure occurrences. Thus, we have maintained some data balancing in terms of seizure and non-seizure EEG epoch ratios for effective training on seizure events. On the other hand, 911 h of EEG data were used to test the performance of our system. The AE model for the encoded representation of the input signal was trained using the training data. The encoded representation of these EEG epochs was used to train the classifier. In order to prevent model overfitting, the 5-fold cross-validation strategy was used for AE training.

Additionally, for the Bonn database, a similar protocol for testing and training was utilized. EEG epochs of 173 samples each were processed with an AE hidden size of 44. Due to the small size of the total dataset, researchers have used from 50% to 90% of the data for training and the rest for testing. The EEG epochs were selected randomly for the two sets. No constraints for data balancing were used, as the seizure and non-seizure EEG epochs are found in equal quantities in this dataset. The training/testing ratio for the BONN dataset is 70% and 30%, respectively, in our case. In addition, test data are devided into 10% validation and 20% test sets. This is not surprising, as this training data size is still smaller than the one that we used for the CHB-MIT dataset.

### 4.2. Performance Evaluation

**Figure 3:**
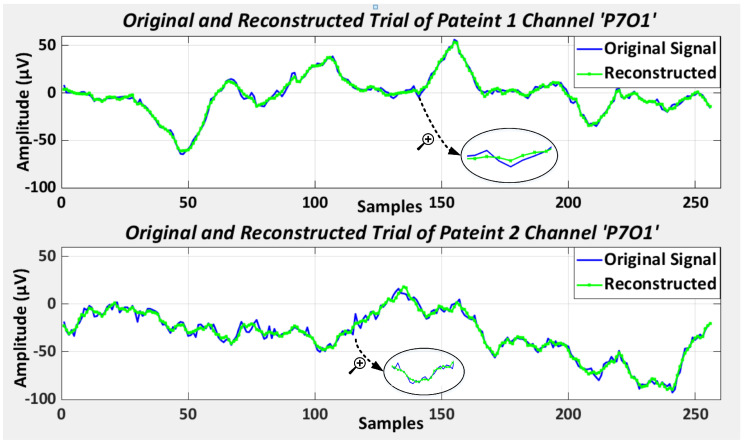
Original and reconstructed EEG epoch during training.

The encoded representation of the EEG signal is characterized in Figure 3 by addressing a random example of the original EEG signal and its reconstruction by decoding. The first figure shows patient 1’s EEG epoch for channel ’P7O1’ of the database, whereas the second signal is the corresponding EEG epoch for the same channel for patient 2. This implies the patient-wise consistency of the methodology. With a mean absolute error of 0.68 and 0.71, respectively, only a minor difference can be seen between the two plots, which infers that encoding the EEG data is a more efficient method for encoded representation as compared to evaluating statistical features.

**Figure 4:**
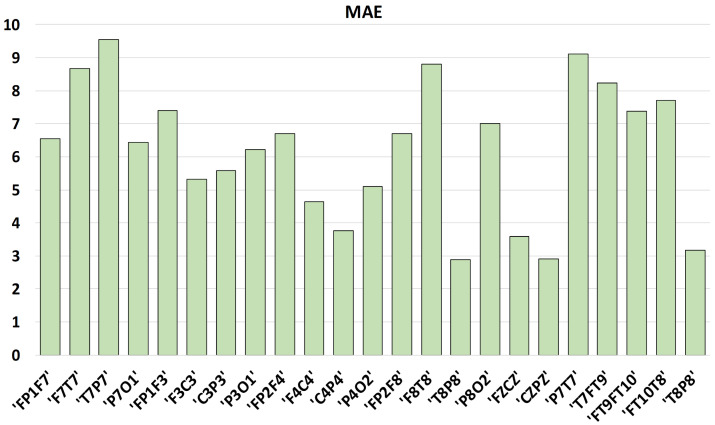
Average MAE of reconstructed signal for each channel with AE hidden size 64.

The average mean absolute error (MAE) calculated from the reconstructed signal is addressed in Figure 4 for each EEG channel. Our goal in this paper is to correctly categorize the data as epileptic or non-epileptic, which is accomplished through encoded representation. Moreover, if a user (for example, a neurologist) needs to analyze the reconstructed signal, our proposed algorithm can be utilized for that purpose as well.

We investigated a diverse range of AE hidden sizes to obtain an optimized feature vector for classifier models. Although kNN obtains the highest classification figures for an AE hidden size 64, it also produces promising results for other AE hidden sizes. SVM, on the other hand, provides the best score for an AE hidden size of 32. Table 2 shows the channel-wise mean testing measures in terms of accuracy, sensitivity, and specificity for all EEG channels. The AE hidden size is 64. All the results for each EEG channel exhibit quality metrics above 94%, which addresses the effectiveness of the proposed methodology. Table 3 encapsulates the same results for AE hidden sizes of 32 using the SVM classifier.

Table 2 and Table 3 correspond to the two flavors (cases) of our method. The first one is the combination of AE with the kNN classifier, providing the best classification results over all classifiers and AE hidden sizes that we experimented with. This conclusion takes into account that all three metrics (accuracy (acc), sensitivity (sen), and specificity (sp)) perform the best or close to the best. This performance can be compared with the second case, which is based on combining AE with an SVM classifier. Here, the accuracy and specificity are even better, but the sensitivity has decreased. This gap in performance in sensitivity shall not be ignored in terms of ranking the overall classification performance due to the equal importance of this metric. Nevertheless, this second case carries merit, as its performance is on the higher side among the state-of-the-art reported approaches, and furthermore, it has low computational costs and memory requirements compared to other state-of-the-art approaches and our kNN-based approach.

Table 4 encapsulates a brief comparison of the obtained results with different classifiers using the average figures of all the EEG channels. kNN achieved the highest results for all three statistical measures using an AE hidden size of 64; this was followed by SVM’s results. The traditional softmax classifier used in deep learning models provides the lowest figures among all the listed classifiers.

**Figure 5:**
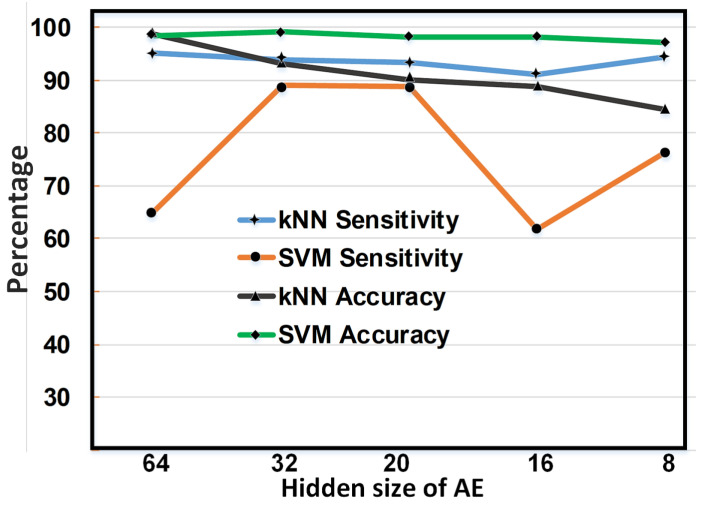
Performance variations with different AE hidden sizes for kNN and SVM classifiers in terms of accuracy and sensitivity.

Figure 5 encapsulates the trends in the performance of SVM and kNN with changes in AE hidden sizes for EEG data. As for the sensitivity, which is an area of concern, kNN maintained its performance above 90%, even for an AE hidden size of 8, which corresponds to only 8 EEG samples out of 256. Only a minor fluctuation can be perceived in Figure 5 for kNN sensitivity. However, the SVM classifier shows instability in terms of sensitivity for changes in the AE’s hidden size. Performance considerations regarding seizure detection accuracy show the opposite trend for both classifiers. SVM is able to catch a glimpse of inconsiderable change in accuracy measures for a decrease in the AE’s hidden size of the EEG signal. Contrarily, kNN evaluates a bit more variation as compared to SVM while categorizing the data for different AE hidden sizes.

**Figure 6:**
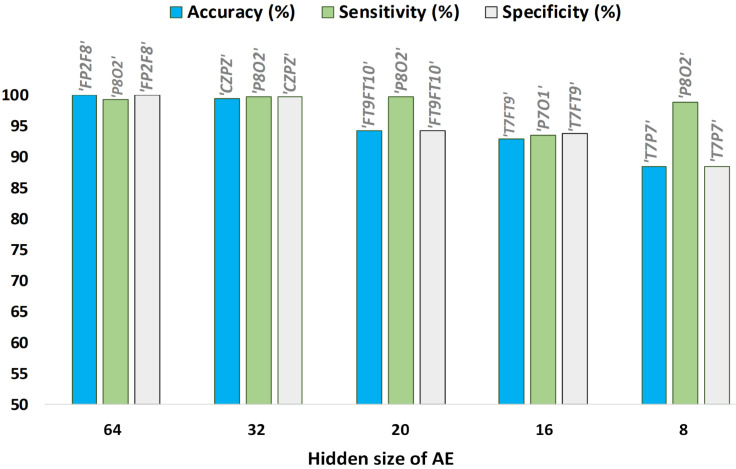
Best classification results of single EEG channel using kNN for various AE hidden sizes.

Figure 6 depicts the quality measures of the proposed seizure detection algorithm according to the AE hidden size. A single EEG channel providing the overall best classification results among all the available channels for a specific AE Hidden size is shown. For AE hidden sizes of 64 and 32, the channel ’P8O2’ provides the highest-quality statistics. However, channel ’FT9FT10’ provides optimum results for 20 samples, channel ’P7O1’ best performs for an AE hidden size of 16, and channel ’T7P7’ yields its best classification performance for 8 samples.

**Figure 7:**
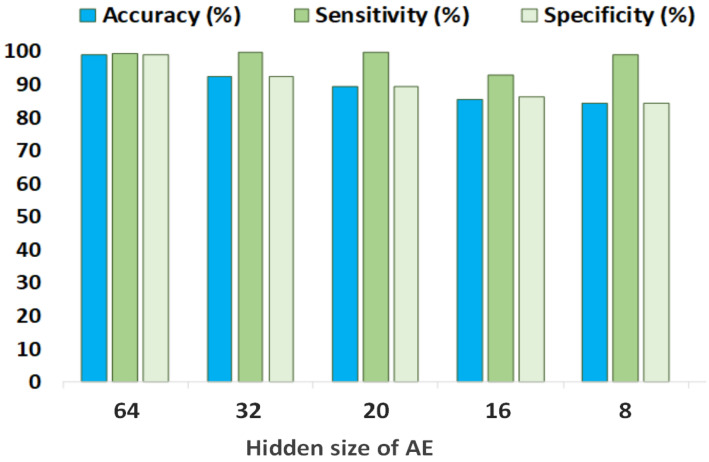
Classification results of channel ’P8O2’ with kNN for different AE hidden sizes.

The classification results obtained by the EEG channel ’P8O2’ for different AE hidden sizes are presented in Figure 7. This channel produces the highest classification figures among all the other available channels of the database for two AE hidden sizes. Seizure detection sensitivity is above 99% for AE hidden sizes of 64 and 32, whereas the minimum sensitivity of this channel is 93%. These statistics also demonstrate the effectiveness of the proposed classification algorithm for a specific EEG channel.

**Figure 8:**
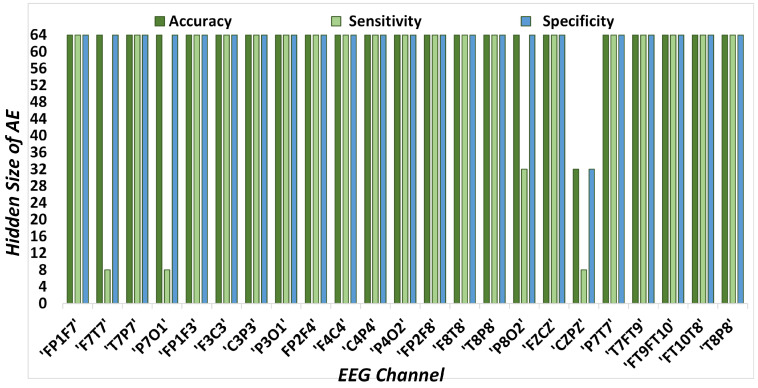
AE hidden sizes providing best accuracy, sensitivity, and specificity with kNN for each channel.

AE hidden sizes providing the best classification results in terms of accuracy, sensitivity, and specificity for each EEG channel are presented in Figure 8. An AE hidden size of 64 produced the highest results in most cases. Accuracy and specificity are the highest when obtained with an AE hidden size of 64 for all the channels. However, for the channels ’F7T7’, ’CZPZ’ and ’P7O1’, the highest sensitivity is achieved using s AE hidden size of 8.

## 5. Discussions

### 5.1. Performance Comparison

To illustrate the effectiveness of the proposed method, the obtained results are compared with the state-of-the-art approaches in Table 5 for the CHB-MIT dataset and Table 6 for BONN database. An exact comparison is challenging as the approaches vary in terms of the number of channels used and the different training and testing scenarios, along with different EEG epoch sizes. Table 5 encapsulates information on different approaches. Those approaches are listed for comparison that classify EEG at the segment level (of intervals 1–30 s) and provide classification results in terms of accuracy, sensitivity, and specificity. Both multi-channel as well as single-channel methods are enlisted. Patient-specific results are also indicated. The training strategy among reported approaches varies from 5-fold and 10-fold cross-validation (CV) to leave-one-out CV (LOO-CV).

Table 5 includes two cases of our proposed approach: one achieving the best performance in terms of average accuracy, sensitivity, and specificity (using kNN classifier) and the other of low computational complexity but providing state-of-the-art classification performance. The computational complexity of the latter approach is analyzed and compared favorably with the reported approaches in the following section. The performances of all approaches are indicated in terms of their reported average classification accuracy, sensitivity, and specificity. Our approach is also presented in terms of its average performance figures for the full training set (i.e., for all EEG channels and for all patients) processed one channel at a time. Individual channel-wise performance for our cases have already been covered in Table 2 and Table 3.

The approach demonstrates its potential for use in extended monitoring/diagnosis in the case of ambulatory patients. After making a regular diagnosis with standard EEG equipment at the hospital, the neurologist is expected to make a smart decision about channel(s) to use (one or more) for a given patient. This can minimize the number of channels (for patient comfort) and resources (for long battery life and form factor) required in the context of a wearable device and still provide top performance in terms of seizure detection.

### 5.2. Computational Complexity

The computational complexity of multi-channel approaches is generally far higher than single-channel approaches, as multi-channel approaches involve preprocessing and feature extraction from many channels. This is supported by [50], which provides a useful comparison of the computational cost of different classifier models for seizure detection. This study shows that the number of EEG channels relates directly to computational costs. It is to be noted that most of the techniques in the literature [1,2,3,4,5,6,7,8,9,10,11,12,13] use multi-channel processing and hence require far more computational resources. This also applies to hardware implementations of [11,30]. Furthermore, it is also shown that for neural networks, the number of layers directly relates to computational costs and memory requirements [50]. It also enlists the SVM classifier as the second model having the least computational and memory requirements.

Our approach, at its basis, is a single-channel approach, which can be extended to multi-channel EEG. However, keeping in view its top classification performance and the application scenario, as discussed earlier, the number of utilized channels may remain confined to one or a few selected channels. Furthermore, the use of a shallow encoder replaces the preprocessing and complex feature extraction stages in other approaches. Thus, the proposed architecture provides lower computational complexity. As part of our hybrid EEG seizure detection approach employing AE, various machine learning algorithms for the classification of seizure and non-seizure EEG signal epochs have been examined. In our proposed method, kNN shows the most promising classification results (for an AE hidden size of 64). However, it requires memory storage and the retrieval of many trained examples, which is not hardware-friendly. The SVM classifier is more attractive for hardware implementation as it eliminates the above issue with comparable second-highest performance results (for an AE hidden size of 32). This is computationally light and still out-competes current approaches in classification performance. For the best-case performance, the average time for processing a 1 s EEG epoch is about 1.6 ms with kNN (with an AE hidden size of 64) and 0.36 ms with SVM (with an AE hidden size of 32) on our platform.

In Table 7, we have compared the computational complexity of the proposed algorithm (single-layer AE with SVM classifier) with some of the recent promising seizure detection approaches. This includes all the single-channel approaches referred to in Table 5 and some multi-channel approaches (both neural network and traditional approaches) as examples to portray the expected computational complexity range. We will focus on the analyzing the computational complexity in the testing/operational mode as training is expected to take place off-line. The training involved in our case is not heavier than other trainable approaches, both with respect to the size of training data as well as the computations involved in our simpler architecture.

Table 7 compares the mathematical operations required for feature extraction (FE) and classification (CLS) for a single EEG epoch. These operations include the number of multiplications and additions required for feature extraction and classification. Our algorithm does not involve any preprocessing. However, it is mentioned in Table 7 if any of the listed methods perform preprocessing. We have, however, excluded their preprocessing stages in computation calculations for simplicity. Furthermore, the tabulated computations correspond to the testing phase only (in the case of training-based approaches). The reason for this choice is the fact that the trained model can be prepared online only once and then used for real-life seizure detection. As illustrated in Table 7, the computational complexity of our proposed seizure detection algorithm is far lower compared to other techniques.

When comparing the basic processing architecture of the proposed seizure detection methodology with existing techniques, it can be seen that our proposed model out-competes the state-of-the-art models in terms of classification performance as well as the computational cost. Some major drawbacks of the existing methods are the pre- and postprocessing units requiring input data transformation and noise addition, as in [17]. These steps are avoided in our proposed method to reduce the required computations. An assessment of our proposed model on two different epilepsy datasets is provided, and EEG segment level results are addressed, which is not provided in [17]. Furthermore, some approaches, such as [9], use convolutional layers in their proposed neural network, which is also computationally expensive compared to a fully connected network. In addition, the proposed seizure detection model uses a shallow architecture instead of deep networks as in [9]. This shallow architecture makes the model computationally efficient. Due to the use of a single-layer model, network parameters become significantly lower compared to deep networks. Consequently, computational and storage requirements to process the data become lower. Moreover, the compression ratio of the existing methods that utilize the encoded representation of EEG data for classification is also low [17]. The proposed model provides a higher compression ratio for low-dimensional encoded representation compared to the existing methods for seizure detection. As a result, the amount of input features for the classifier model decreases, making the model computationally efficient, and convergence of classifier becomes easier. Thus, practically, it has a higher potential for hardware implementation in wearable devices.

Our proposed approach can be scaled up to a multi-channel approach, as it can be seen as channel-wise modular. This would mean adding a final ensembling stage that can combine the seizure detection results obtained at the individual channel levels. Table 7 shows that in our case, the per-channel computational effort is the lowest. It can be deduced that the total computational complexity after scaling up to multiple channels will remain lower compared to other approaches with an equivalent number of channels. However, this effort is beyond the scope of this research, which is limited to demonstrating the potential towards seizure activity detection in a single channel with high accuracy and low complexity.

Preprocessing was not included as the dataset used in our study is already preprocessed for the removal of artifacts. Furthermore, our approach does not involve feature extraction.

## 6. Conclusions

We have proposed a low-complexity epileptic seizure detection algorithm with potential applications in wearable health systems. The approach is based on single-channel analysis with the potential to modularly build a multi-channel seizure detector. The algorithm design is simpler as it does not involve design complexities related to the feature extraction stage, such as feature selection, feature raking, etc. A shallow autoencoder with a single encoder layer has been used to obtain various encoded representations, which are classified using a conventional classifier. We have compared our hybrid approach performance with both deep learning models and traditional techniques. Our approach outperforms the state-of-the-art methods with regard to classification accuracy, sensitivity, and specificity, with classification sensitivity at 99% using a kNN classifier, which provides the most promising results. The relatively low-complexity SVM classifier also out-competes the existing approaches and is more hardware-implementation-friendly.

## Figures and Tables

**Table 1:** AE Hidden size with corresponding data reduction and arithmetic operations required for 256 actual samples.

AE Hidden Size	Data Reduction (Compression Ratio)	Arithmetic Operations (AE)	Arithmetic Operations (SVM)
64	75.00% (4)	32,704	1057
32	87.50% (8)	16,352	577
20	92.18% (12.8)	10,220	397
16	93.75% (16)	8176	337
8	96.87% (32)	4088	217

**Table 2:** Channel-wise average accuracy, sensitivity, and specificity of kNN classifier on the CHB-MIT database with AE hidden size of 64 (Compression Ratio = 4).

Channel	Accuracy (%)	Sensitivity (%)	Specificity (%)
’FP1F7’	98.17	95.04	98.28
’F7T7’	99.46	94.17	99.46
’T7P7’	96.26	95.15	96.26
’P7O1’	99.32	94.47	99.32
’FP1F3’	98.28	95.20	98.98
’F3C3’	98.60	95.16	98.60
’C3P3’	98.72	95.07	98.72
’P3O1’	99.20	94.59	99.20
’FP2F4’	99.04	95.04	99.04
’F4C4’	99.35	96.39	99.35
’C4P4’	99.21	94.81	99.38
’P4O2’	99.05	96.40	99.05
’FP2F8’	99.90	94.59	99.90
’F8T8’	98.58	95.40	98.58
’T8P8’	98.17	94.47	98.17
’P8O2’	98.85	99.29	98.85
’FZCZ’	98.65	95.46	98.97
’CZPZ’	99.79	94.33	99.79
’P7T7’	96.61	94.74	96.61
’T7FT9’	96.36	95.12	96.36
’FT9FT10’	97.26	94.94	97.26
’FT10T8’	99.32	96.13	99.32
’T8P8’	98.17	94.47	98.86

**Table 3:** Channel-wise average accuracy, sensitivity, and specificity of SVM classifier on the CHB-MIT database with AE hidden size of 32 (compression ratio = 8).

Channel	Accuracy (%)	Sensitivity (%)	Specificity (%)
’FP1F7’	99.73	86.83	99.74
’F7T7’	98.91	91.81	98.92
’T7P7’	99.11	91.67	99.11
’P7O1’	99.30	89.64	99.31
’FP1F3’	99.59	92.15	99.60
’F3C3’	99.39	90.47	99.40
’C3P3’	99.19	96.10	99.19
’P3O1’	98.88	87.75	98.89
’FP2F4’	99.33	91.79	99.35
’F4C4’	99.57	93.21	99.58
’C4P4’	99.47	93.80	99.48
’P4O2’	99.46	92.52	99.47
’FP2F8’	99.54	88.21	99.55
’F8T8’	99.69	95.41	99.70
’T8P8’	99.73	86.83	99.74
’P8O2’	99.67	94.93	99.67
’FZCZ’	99.04	95.27	99.04
’CZPZ’	99.18	29.75	99.31
’P7T7’	99.01	91.63	99.01
’T7FT9’	98.52	90.84	98.53
’FT9FT10’	99.57	94.34	99.57
’FT10T8’	98.46	93.76	98.47
’T8P8’	99.02	89.32	99.31

**Table 4:** Average classification results of CHB-MIT database for various AE hidden sizes with different classifiers.

AE Hidden Size	Classifier	Accuracy (%)	Sensitivity (%)	Specificity (%)
64	kNN	98.60	95.23	98.90
	SVM	98.38	65.29	99.39
	QDA	87.47	47.63	88.71
	NB	92.83	58.23	93.05
	DT	89.96	39.17	91.05
	Softmax	68.31	51.24	61.70
32	kNN	93.08	94.01	93.09
	SVM	99.29	89.03	99.30
	QDA	80.10	60.09	80.12
	NB	82.38	46.90	82.96
	DT	93.99	43.42	94.07
	Softmax	78.50	73.82	79.98
20	kNN	90.00	93.42	90.00
	SVM	98.17	88.89	98.18
	QDA	86.34	61.35	86.37
	NB	82.36	52.32	83.10
	DT	94.28	43.26	94.36
	Softmax	71.63	53.36	74.10
16	kNN	88.77	91.15	89.36
	SVM	98.10	61.79	99.06
	QDA	90.85	43.10	91.98
	NB	90.52	38.36	91.65
	DT	92.55	35.65	93.79
	Softmax	81.62	42.86	72.60
8	kNN	84.47	94.42	84.45
	SVM	97.24	76.16	97.27
	QDA	92.47	43.57	92.54
	NB	88.06	48.23	88.63
	DT	94.97	35.69	95.06
	Softmax	53.68	58.90	48.86

**Table 5:** Comparison of classification results for CHB-MIT database.

Ref., Year	No. of Channels	Method	Acc (%)	Sen (%)	Sp (%)	Evaluation & Data Used
[7], 2021	Multiple	EMD, DWT, CSP & multi-SVM fusion	97.49	97.34	97.50	Tr: 31.6 h; Te = 945.3 h
[38], 2021	23	DWT, OMP, SFFS & SVM	97.09	96.81	97.26	177 h; 5-fold CV
[3], 2020	21	Spectral, temporal, CE-stSENet	95.96	92.41	96.05	For each patient, 1.11 h inter-ictal and all ictal data
[12], 2020	23	LDA & ANN	95.11	91.15	95.16	42.8 h; Tr: 25%; Te: 75% (PS)
[4], 2020	22	30 Statistical features & XGB	86.27	80.32	92.22	5-fold CV (PS)
[8], 2020	Multiple	24 Statistical features & SVM	94.50	-	-	-
[27], 2020	23	DWT & ANN	95.3	97.2	93.5	Tr: 208.3 h, Te: 23.3 h
[11], 2020	2	Statistical features &RF	-	96.60	92.50	Tr: 70%, Te: 30%
[25], 2020	23	LSB, Spectral energy &SVM	91.70	95.00	-	Tr: 80%, Te: 20% (PS)
[9], 2019	23	SAE, Deep CNN, FC & SVM	92.00	95.00	90.00	LOO-CV (PS)
[5], 2019	23	Deep CNN, Dense & SoftMax	98.05	90.00	91.65	Test on 1 patient, train rest
[47]-2019	23	Deep CAE, FC & SoftMax	93.97	-	-	210.7 h; 5 fold CV
[32], 2019	Multiple	Statistical features, PCA, SVM & NB	95.63	95.70	96.55	Tr: 50%, Te = 50% (PS)
[6], 2018	Multiple	Statistical features, Change point detection	-	96.00	-	LOO-CV for each seizure (PS)
[33], 2018	Multiple	FFT, Deep CNN, FC & SoftMax	96.10	-	-	LOO-CV for each seizure
[24], 2018	23	EMD dictionary & SVM	92.90	94.30	91.5	(PS)
[16], 2018	21	Deep CNN & SoftMax	-	87.95	86.50	80% inter-ictal, 20% ictal, LOO-CV
[21], 2017	Multiple	FFT, DWT, ST & Channel aggregate	-	91.40	86.00	-
[20], 2016	23	Poincare section, PCA, LDA & NB	94.69	89.10	94.80	Tr: 50%, Te: 50% (PS)
[10], 2021	1	Band energy & SVM	-	87.60	88.00	(PS)
[29], 2017	1	DWT & SVM	96.80	72.00	98.11	0.4 h iner-ictal, all ictal, (PS)
[22]-2015	1	DWT, PCA & SVM	93.96	75.75	98.66	916 h with 10 fold CV
***Proposed:***						
Optimal (Single-channel mean)	1	AE & SVM; 32 encoded features	99.29	89.03	99.30	Tr: 5 h; Te: 911 h
Best Results (Single-channel mean)	1	AE & kNN; 64 encoded features	98.60	95.23	98.90	Tr: 5 h; Te: 911 h

Acc: accuracy; Sen: sensitivity; Sp: specificity; PS: patient-specific; Tr: training data; Te: test data; h: Hours; CV: cross-validation.

**Table 6:** Comparison of classification results for BONN database.

Ref., Year	Method	Data	Acc (%)	Sen (%)	Sp (%)	Evaluation
[1], 2021	CNN–RNN	A vs. E	99.61	99.81	99.43	10 fold CV
[38], 2021	DWT & OMP with SVM	A vs. E	99.50	98.95	100	5-fold CV
[40], 2021	CNN & sigmoid	A vs. E	93.00	93.00	93.00	80% train, 20% test
[23], 2021	RPS average energy & SVM	A vs. E	99.50	99.00	100	10-fold CV
[42], 2019	Symlets wavelets, PCA & SVM	A vs. E	100	-	-	10-fold CV
[41], 2017	Local mean decomposition & SVM	A vs. E	100	100	100	50% train, 50% test
This work	44 AE features & SVM	A vs. E	99.64	99.71	99.56	70% train, 30% test
[1], 2021	CNN–RNN	A vs. E	99.46	99.17	99.22	10-fold CV
[38], 2021	DWT & OMP with SVM	B vs. E	99.75	98.60	100	5-fold CV
[23], 2021	RPS average energy & SVM	B vs. E	98.50	97.00	100	10-fold CV
[42]-2019	Symlets wavelets, PCA & SVM	B vs. E	100	-	-	10-fold CV
This work	44 AE features & SVM	B vs. E	97.71	99.30	96.71	70% train, 30% test
[1], 2021	CNN–RNN	A vs. E	99.51	99.31	99.43	10-fold CV
[38], 2021	DWT & OMP with SVM	C vs. E	99.00	98.89	98.89	5-fold CV
[23], 2021	RPS average energy vs. SVM	C vs. E	98.00	97.00	99	10-fold CV
[42], 2019	Symlets wavelets, PCA vs. SVM	C vs. E	98.4	-	-	10-fold CV
This work	44 AE features & SVM	C vs. E	99.42	99.86	98.96	70% train, 30% test
[1], 2021	CNN–RNN	A vs. E	99.82	99.68	99.82	10-fold CV
[38], 2021	DWT & OMP with SVM	D vs. E	99.50	100	99.16	5-fold CV
[40], 2021	CNN & sigmoid	D vs. E	88.50	92.00	85.0	80% train, 20% test
[23], 2021	RPS average energy & SVM	D vs. E	97.50	95.00	100	10-fold CV
[42], 2019	Symlets wavelets, PCA & SVM	D vs. E	98.1	-	-	10-fold CV
[41], 2017	Local mean decomposition & SVM	D vs. E	98.10	98.80	97.40	50% train, 50% test
This work	44 AE features & SVM	D vs. E	98.77	99.71	97.85	70% train, 30% test

**Table 7:** A comparative analysis of computational cost with recent seizure detection methods.

Ref., Year	Channels	Preprocessing	Methodology	Multiplications (FE + CLS)	Additions (FE + CLS)
[5], 2019	23	Nil	7-layer CNN, dense & SoftMax *	6161.7 K (6161.4 K + 0.35 K)	6144.5 K (6144.5 K + 0.04 K)
[16], 2018	21	Polar projection & FFT	11 layers CNN & SoftMax *	777.5 K (777.2 K + 0.35 K)	691.2 K (691.1 K + 0.04 K)
[10] ***, 2021	1	Nil	Filter bank, (10 bands), band energy & SVM	235,540 (235.5 K + 0.02 K)	235.5 K (235.5 K + 0.02 K)
[22], 2015	1	Nil	7 DWT levels & SVM	130,858 (130.8 K + 0.04 K)	127.7 K (127.7 K + 0.06 K)
[36], 2021	1	Nil	OMP & Binary classifier	475,436.3 K	474,817.1 K
[29] **, 2017	1	Nil	7 DWT levels & SVM	391.1 K	390.0 K
[17], 2018	1	Noise addition & Z-score	AE & LR	51.7 K (51.2 K + 0.5 K)	51.4 K (51 K + 0.4 K)
[40], 2021	1	Filter bank & Recurrence plots	7-layer CNN & Sigmoid *	5037.3 K (5037.1 K + 0.17 K)	4023.8 K (4023.8 K + 0.02 K)
Proposed	1	Nil	Single-layer AE (sparsity = 8) & SVM	8.5 K (8.1 K + 0.34 K)	8.3 K (8.1 K + 0.2 K)

* We used 10 Taylor series approximations for the exponent. ** Classifier operations are not included in this paper as complete information is missing. *** Number of filter coefficients are not mentioned, so we used the value from [51]. feature extraction (FE); classification (CLS); K: Kilo (1000).

## Data Availability

Not applicable.

## Abbreviations

The following abbreviations are used in this manuscript:

AE	Autoencoder
ASIC	Application Specific Integrated Circuit
CAE	Convolutional Autoencoder
CNN	Convolutional Neural Network
CSP	Common Spatial Patterns
CR	Compression Ratio
CV	Cross Validation
CLS	Classification
LOO-CV	Leave One Out Cross Validation
CHB-MIT	Children Hospital Boston, Massachusetts Institute of Technology
DT	Decision Tree
DWT	Discrete Wavelet Transforms
EEG	Electroencephalogram
EMD	Empirical Mode Decomposition
FE	Feature Extraction
GARCH	Generalized Autoregressive Conditional Heteroscedasticity
kNN	k Nearest Neighbour
LDA	Linear Discriminant Analysis
LSP	Lomb-Scargle Periodogram
LSTM	Long Short Term Memory
MAE	Mean Absolute Error
NB	Naive Bayes
OMP	Orthogonal Matching Pursuit
PCA	Principal Component Analysis
QDA	Quadratic Discriminant Analysis
QDNN	Quantized Deep Neural Networks
RF	Random Forest
SVM	Support Vector Machine
WHO	World Health Organization
